# Comparison between *Lactobacillus rhamnosus* GG and LuxS-deficient strain in regulating gut barrier function and inflammation in early-weaned piglets

**DOI:** 10.3389/fimmu.2022.1080789

**Published:** 2022-12-08

**Authors:** Zhaoxi Deng, Jinyan Dai, Yusen Wei, Yanfei Ma, Yingying Mao, Jinzhi Zhang, Weidong Hua, Haifeng Wang

**Affiliations:** ^1^ Experimental Livestock Farm of Animal Husbandry and Veterinary Research Institute, Zhejiang Academy of Agricultural Sciences, Hangzhou, China; ^2^ College of Animal Science, Ministry of Education (MOE) Key Laboratory of Molecular Animal Nutrition, Zhejiang University, Hangzhou, China; ^3^ Laboratory Animal Center, Sichuan University, Chengdu, China

**Keywords:** *Lactobacillus rhamnosus* GG, *luxS* mutant, quorum sensing, early-weaning, piglets, intestinal barrier function

## Abstract

**Background:**

Early weaning-induced stress impairs the intestinal barrier function and adversely affects the health of piglet. Probiotics can be used to prevent and treat various intestinal diseases. *Lactobacillus rhamnosus* GG (LGG) has an LuxS/AI-2 quorum sensing (QS) system that senses environmental changes through chemical signaling molecules. The aim of the study was to explore whether *luxS* mutant affects the protective role of LGG in the gut barrier of weaned piglets by comparing the *luxS* mutant (ΔluxS) with its wild-type (WT).

**Methods:**

Newborn piglets were orally administered with WT and ΔluxS at dosage of 10^9^ CFU, respectively. Accordingly, newborn piglets in the Con group were orally administered with PBS. Piglets were weaned on day 21 and euthanized on day 24, three days following weaning.

**Results:**

Supplementation of ΔluxS in advance significantly boosted the relative abundances of healthy microbes (including *Catenibacterium*, *Eubacterium*, *Lachnospiraceae* and *Bifidobacterium*). WT and ΔluxS maintain intestinal barrier function mainly by promoting intestinal villus to crypt ratio (VCR), occludin protein expression and mucus secretion (*P*<0.05). Furthermore, LGG reduces pro-inflammatory mediators by inhibiting TLR4 and MAPK signal transduction (*P*<0.05).

**Conclusion:**

Both WT and ΔluxS were shown to resist weaning stress by enhancing the intestinal barrier function of piglets. It has to be said that the ability of ΔluxS to maintain intestinal tissue morphology and promote mucus secretion significantly decreased compared with that of WT.

## Background

Early weaning helps to shorten the reproductive cycle and increase the birth number of sows, which has been extensively used in a variety of livestock and poultry (1, 2). However, newborn piglets are characterized by imperfect digestive system, unstable intestinal microbiota, and lack of innate immunity, those are prone to cause a variety of intestinal diseases, including intestinal inflammation and diarrhea (3, 4). The high mortality rate induced by early weaning is an important factor restricting the vigorous development of animal husbandry.

Probiotics, which are live cultures of beneficial bacteria, are generally an effective tool for controlling the dynamics of gut microbiota. *Lactobacillus rhamnosus* GG (LGG) is a probiotic from the intestinal tract of healthy individuals, which maintains gut health by enhancing barrier integrity and the stability of the microbiota (5, 6). Research has showed that LGG supplementation in diets relieved the diarrhea of weaned piglets challenged by RV *via* preventing virus multiplication and promoting the jejunal mucosal barrier function (7).

The intestinal epithelial barrier function, including biological, physical, chemical, and immune barriers, is critical to animal gut health (8). The gut microbiota constitutes the biological barrier that prevents pathogen from colonizing the intestine and contributes to the maturation of the gastrointestinal tract and immune system (9). The physical barrier of the intestinal epithelium confers the direct characteristic of the selective permeability to the gut, especially the intestinal tight junction. Tight junctions are the rate-limiting step in epithelial transport and a major determinant of mucosal permeability (10). The intestinal tight junction barrier consists of protein complexes including zonula occludens (ZO), occludin and claudin family members (11). Probiotics stimulate intestinal epithelial cells to express mucin, promote mucus secretion, form a protective layer between the mucosa and microorganisms, and enhance the intestinal mucosal barrier function (12). Supplementation with LGG resists barrier injury caused by IFN-γ by restoring occludin and ZO-1 to normal levels in human gut cells (13).

Quorum sensing (QS) is a way that bacteria use signaling molecules to communicate information, which can influence bacterial toxicity, luminescence and biofilm information (14). The LuxS/AI-2 quorum sensing system exists in LGG to mediate bacterial intra- or inter-species information exchange. As AI-2 produced by one species could affect gene expression in another, this feature makes AI-2 to act an excellent candidate for mediating cell-cell interactions in the mammalian gut. In a previous study we have found that deficiency of the LuxS/AI-2 QS system reduced LGG AI-2 activity, interbacterial adhesion and biofilm formation (15). Based on studies of bacterial cell interactions in laboratory systems, it is likely that the exchange of these signals plays a critical role in the dense bacterial communities exist in the mammalian gut (16). Whether the LuxS/AI-2 QS system also affect bacterial probiotic effects in the animal gut by regulating some functions of LGG through signaling molecules? We focus on the effects of LGG wild-type (WT) and luxS-deficient strain (ΔluxS) on the growth performance, intestinal barrier function and immune system of piglets on third day post weaning by oral administration to newborn piglets, aiming to explore the regulatory effect of the LuxS/AI-2 QS system on weaning stress syndrome in piglets.

## Materials and methods

### Preparation of freeze-dried power

*Lactobacillus rhamnosus* GG (LGG, ATCC 53103) and its ΔluxS mutant were shared by Dr. J.P. van Pijkeren from the University of Wisconsin-Madison. The activated wild-type (WT) and luxS-deficient strain (ΔluxS) of LGG were spread in De Man Rogosa and Sharpe (MRS) solid medium containing streptomycin (100 μg/mL) and cultured anaerobically at 37°C for 48 h. A single colony was picked with a sterile inoculation loop, inoculated into MRS liquid medium, and cultured anaerobically at 37°C for 16 h until LGG with streptomycin resistance was selected. LGG was then anaerobically grown in MRS medium at 37°C for 16 h, and centrifuged at 5000 g for 10 min to discard the supernatant. LGG was resuspended in reconstituted skimmed milk and freeze-dried under vacuum for 48 h. Plate counting detected 109 CFU/g bacteria in the powder. During administration, the bacterial powder that stored in plastic bags at 4 °C was dissolved in PBS for each piglet.

### Animals and treatments

**Figure 1:**
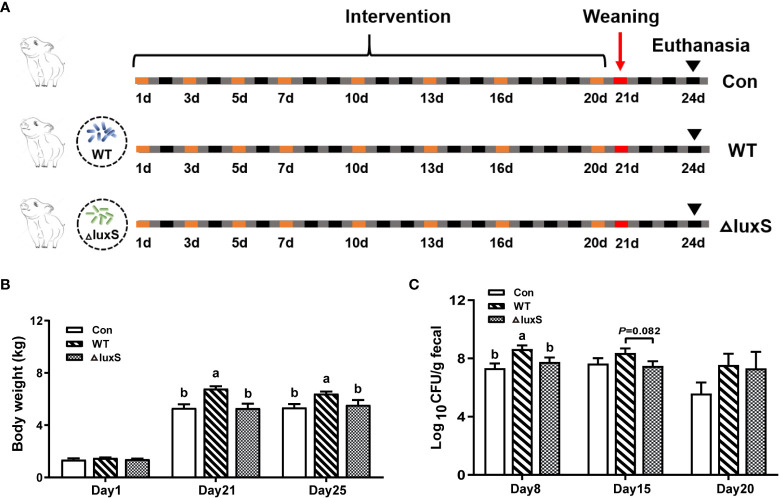
Design of piglet experiments and bacterial colonization in feces. **(A)** Experiment design and workflow. Piglets were randomly divided to 3 groups (6 piglets/group). The WT and ΔluxS groups were orally inoculated with 2 mL of LGG wild strain (WT) and *luxS* mutant strain (ΔluxS), respectively, for 3 weeks (equivalent to 10^9^ CFU/mL, shown as orange box) before weaning on day 21 (red box). Slaughter sampling was performed 3 days after weaning (black arrows). Con group was orally inoculated with PBS. **(B)** The body weights of piglets. **(C)** The colonization of *Lactobacillus* in feces at different time points. Feces were collected on days 8, 15 and 20 for bacterial counts. Statistical significance performed using one-way analysis of variance for multiple comparisons. Mean values not sharing common letters are significantly different (*P*<0.05). Values are shown as mean ± SEM, n=6.

Eighteen newborn piglets with similar body weight were selected and divided randomly into 3 groups with 6 piglets in each group (**Figure 1A**). Newborn piglets were orally administered with 2 mL of WT and ΔluxS on days 1, 3, 5, 7, 10, 13, 16 and 20, respectively (equivalent to 10^9^ CFU bacteria). Piglets in the control group were administered orally with 2 mL sterile PBS. Piglets were weaned on day 21 and euthanized 3 days after weaning. Body weights were recorded at the beginning (the 1th day), the weaning (the 21th day) and the slaughter day (the 25th day) in this work. Feces were collected on days 8, 15 and 20. The schematic drawing of the experimental flow chart can be found in **Figure 1A**. This animal procedure was approved by the Animal Care and Use Committee of Zhejiang University (ETHICS CODE Permit no. ZJU20170529).

### Counting of *Lactobacilli* excretion in feces

The number of *Lactobacilli* excreted in feces was determined by MRS agar plates containing streptomycin. The feces collected on days 8, 15 and 20 were resuspended in PBS and serially diluted. The dilutions of various concentrations were grown on MRS agar plates overnight at 37°C, followed by colony enumeration.

### 16S rRNA Sequencing and analysis

Microbial DNA was extracted from ileum contents using the DNA extraction Kit (Omega Bio-tek, USA) following the manufacturer’s protocols. The total DNA was inspected by NanoDrop 2000 (Thermo Fisher, USA) and 1% agarose gel electrophoresis. The 16S rRNA genes were amplified using the specific primer for V3-V4 region (17). And the bacterial 16S rRNA gene was amplified on the Illumina platform using a double-indexed, paired-end sequencing strategy as described previously (18). KAPA HiFi Hotstart Ready Mix PCR kit is used for PCR amplification and product purification. Subsequently, amplicons were multiplexed and sequenced on the Illumina MiSeq platform at Majorbio Bio-Pharm Technology Co. Ltd. (Shanghai, China). The raw sequenced reads were demultiplexed, quality-filtered by fastp version 0.20.0 (19, 20). Operational taxonomic units (OTUs) were defined by clustering 97% identity sequences, and chimeric sequences were identified and removed using UPARSE (Version 7.1) (21). Classification of representative reads for each OTU was carried out utilizing the RDP Classifier algorithm with the confidence threshold of 70%. Taxonomy was assigned to the OTU referred to the SILVA database. Raw sequence data were deposited into the NCBI Sequence Read Archive (SRA) database (PRJNA856449).

### Serum biochemical analysis

The blood was processed to obtain serum by centrifugation at 3000 g for 10 min at 4°C. The content of diamine oxidase (DAO), intestinal trefoil factor (ITF), TNF-α and IL-6 in the serum were determined using commercially available ELISA kits (Shanghai FANKEL Industrial Co., Ltd, China). In addition, antioxidant indexes in serum were measured using commercially available kits (Nanjing Jiancheng Inc, China). These indexes included activities of superoxide dismutase (SOD), glutathione peroxidase (GSH), and total antioxidant capacity (T-AOC). All steps were done according to manufacturer protocols.

### Morphological analysis of intestinal tissues

Hematoxylin-eosin (H&E) staining was performed as previously described to examine morphology and structure of the gut (22). Briefly, after being taken out from the 4% paraformaldehyde solution, the intestinal tissues of the piglet were washed and embedded in wax. Paraffin blocks were cut into 3-µm thick sections and subjected to deparaffinization and a series of dehydration processes. Subsequently, sections were immersed in a gradient of alcohol and stained with H&E. Intestinal morphology of the samples were characterized using an optical microscopy (Olympus Corporation, Japan) at 100× magnification.

### Scanning electron microscope

We obtained electron microscopy images as described previously (23). Fresh ileal tissue samples were fixed with 2.5% glutaraldehyde in 0.1 M phosphate buffer for 3 h and then washed twice in the same buffer for 10 min., and post-fixed with 1% osmium tetroxide for 1 h. Subsequent dehydration in varying grades of ethanol is required (20 min each; 30%, 50%, 70%, 90%, 95% and 100% v/v). After the samples were dried with a critical point dryer, the sections were observed with a scanning electron microscope (Hitachi SU-8010, Japan).

### Alcian blue and immunofluorescence staining

The colons were gently separated and immediately stored in Carnoy’s fixative for Alcian blue staining (24). The sample was embedded in paraffin and then was cut into-5μm sections and deposited on the glass slides. Tissue sections were preheated at 60°C for 10 min, immersed in xylene at 60°C for 10 min, and treated with gradient ethanol (100%, 90%, 70%) for deparaffinization. After being stained with Alcian Blue, the samples under glass slide were observed with an Olympus BX63 upright fluorescence microscope equipped with Image J software to measure the number of goblet cells and mucus thickness (24). Anti-Muc2 anti-body immunofluorescence staining was used to show the internal condition of the colon directly. For immunofluorescence staining, tissue sections were covered with primary antibody at 1:200 dilution (Muc2, Biorbyt, USA) at 4°C overnight. After washed three times in PBS for 10 min, the slides were incubated with diluted secondary antibody. Finally, the slides are mounted with a mounting medium containing 4′,6-diamidino-2-phenylindole (DAPI) (25).

### RNA extraction and quantitative real-time PCR

Considering that the colon secretes mucus and covers the surface of intestinal epithelial cells to form a mucus barrier, the colonic mucins expression was tested by quantitative real-time PCR to (26). Total RNA was extracted from the intestinal segments using TRIzol reagent (Tiangen, China) following manufacturer’s instructions. RNA concentration and quality was determined by Nanodrop 2000. Finally, quantitative real-time PCR was performed in a Bio-Rad CFX96 real time PCR machine with the use of SYBR Green Supermix (Takara, Japan) detection protocol. Reaction program was as follows: the mixtures were incubated at 95°C for 5 min, followed by 40 cycles of 5 s at 95°C, 20 s at 60°C, and finally 30 s at 60°C. Melt curves were obtained by increasing the temperature from 65°C to 95°C in 0.5°C increment for 10 s. GAPDH was used as the housekeeping gene. Raw data were analyzed using the 2^-ΔΔCt^ method. The list of primers was available in **Supplementary Table S1**.

### Western blotting analysis

Approximately 50 mg of ileum tissue was homogenized in lysis buffer supple-mented with protease and phosphatase inhibitors (Keygen Biotech, China) and centrifuged at 12,000 g for 10 min at 4°C. Total protein concentrations were detected with a bicinchoninic acid (BCA) protein assay (Keygen Biotech, China). Western blot assay was conducted as previously described using a standard protocol (27). In short, the extracted proteins were resolved on 10% or 12% sodium dodecyl sulfate-polyacrylamide gel electrophoresis gels and transferred to polyvinylidene fluoride (PDVF) membranes. Then incubation with primary antibodies was carried out over night at 4°C. Membranes were then washed and incubated with suitable secondary antibodies. The primary antibodies included anti-claudin-3, anti-occludin, anti-zonula occludens 1 (ZO-1) (Abcam, Cambridge, UK), anti-GAPDH, anti-p38, anti-phospho-p38 (p-p38), anti-extracellular signal-regulated kinase (anti-ERK), anti-p-ERK, anti-TLR4 and anti-IL-1β (Cell Signaling Technology, USA; 1:1000). The secondary antibody was HRP-conjugated goat anti-rabbit IgG (Abbkine, Beijing; 1:5000). Afterwards, the ultrahyper-sensitive ECL chemiluminescence kit (Beyotime Biotechnology, China) was finally used to detect the protein. The target protein bands were visualized with a gel-documentation system and analyzed with Image J software. The density values of bands were corrected by subtraction of the background values.

### Statistical analysis

Data analyses were generated by one-way ANOVA using the GraphPad Prism version 8 Software. Data are presented as the mean ± SEM. Mean without sharing a common letter differ, *P*<0.05. *P*<0.05 was considered as significant and 0.05< *P*<0.1 was considered as a tendency.

## Results

### Growth performance and the colonization of *Lactobacillus* in the gut

The experimental design and workflow are shown in **Figure 1A**. Piglets increased their average weight from 1.36, 1.48 and 1.40 kg at initial for the Con, WT and ΔluxS groups (**Figure 1A**) to 5.34, 6.41 and 5.54 kg, respectively. The weight of piglet in the WT group was significantly higher than that in the Con and ΔluxS groups on the 21st day (weaning day) and three days after weaning (25 days of age) (*P*<0.05, **Figure 1B**). The number of WT strain excreted in the feces was significantly higher on days 8 (*P*<0.05) and had a higher trend on day 15 (*P*=0.082) than that of the ΔluxS strain. No significant difference was observed between the experimental groups on days 20 (**Figure 1C**).

### The microbial community of ileum contents

**Figure 2:**
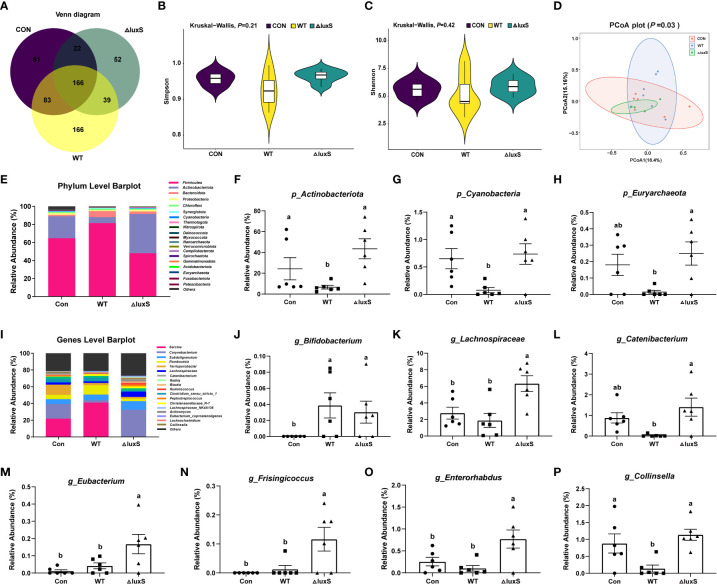
The high-throughput sequencing of 16S rRNA gene in ileum contents. **(A)** Venn disgrams for bacterial OTUs compositions. **(B, C)** α diversity analysis. α diversity was tested by Kruskal-Wallis. **(D)** β diversity analysis. **(E)** Comparison of the phylum level. **(F-H)** Differential bacteria at the phylum level. **(I)** Comparison of the genus level. **(J-P)** Differential bacteria at the genus level. Statistical significance performed using one-way analysis of variance for multiple comparisons. WT and ΔluxS groups were orally inoculated with 2 mL of LGG wild strain and *luxS* mutant strain, respectively, for 3 weeks before weaning on day 21. Con group was orally inoculated with PBS. Mean values not sharing common letters are significantly different (*P*<0.05). Values are shown as mean ± SEM, n=6.

Venn diagram showed that there were 51, 166 and 52 unique taxa in the Con, WT, ΔluxS groups, respectively (**Figure 2A**). No significant differences were found in α-diversity such as Simpson index and Shannon index among the three groups (**Figures 2B, C**). The β-diversity analysis showed that no significant differences were found within and between the treatment groups, indicating that the structure of the microbial community is relatively stable (**Figure 2D**). We further compared the abundance of bacterial taxa at the phylum and genus level in piglets. The *Firmicutes* and *Actinobacteria* constituted the two predominant phyla in the ileal microbiota of the piglets (**Figure 2E**). The relative abundance of *Actinobacteria* and *Cyanobacteria* was significantly lower in the WT group than in the other two groups (*P*<0.05). The relative abundance of *Euryarchaeota* was significantly lower in the WT group than in the ΔluxS group and in the Con group (*P*<0.05), while there was no significant differences between the ΔluxS and the Con group (**Figures 2F-H**). At the genus level, the relative abundance of *Bifidobacterium* in the Con group was significantly less than the WT and ΔluxS groups (*P*<0.05, **Figure 2J**). The relative abundances of *Lachnospiraceae*, *Eubacterium*, *Frisingicoccus* and *Enterorhabdus* in the ΔluxS group were significantly higher than the other two groups (*P*<0.05). The relative abundances of *Catenibacterium* and *Collinsella* were significantly increased in the ΔluxS group than in the WT group (*P*<0.05, **Figures 2I-P**).

### Intestinal barrier function

**Figure 3:**
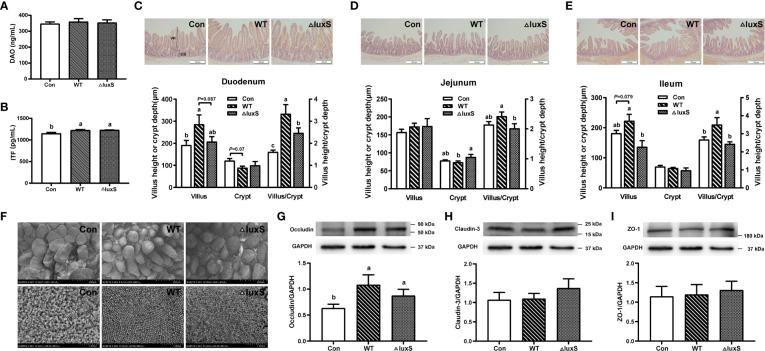
Intestinal physical barrier function in weaned piglets. **(A, B)** DAO and ITF activity in serum of piglet. DAO, diamine oxidase; ITF, intestinal trefoil factor. Intestinal morphology of the duodenum **(C)**, jejunum **(D)** and ileum **(E)** of piglets, scale bars: 100 μm. The villi heights and crypts depths of intestinal segments were measured using Image J software based on at least 25 representatives. The villus length was measured from the villus tip to the villus-crypt junction. The crypt depth was defined as the distance from the base of the crypt to the crypt opening. **(F)** Scanning electron microscopy visualization of piglet ileum. The upper image is of intestinal villi (200 μm) and the lower image is of microvilli (1 μm). The expression of tight junction protein in ileum was evaluated with western blot, including occluding **(G)**, claudin-1 **(H)** and ZO-1 **(I)**. The protein bands were normalized to the intensity values of GAPDH. WT and ΔluxS groups were orally inoculated with 2 mL of LGG wild strain and *luxS* mutant strain, respectively, for 3 weeks before weaning on day 21. Con group was orally inoculated with PBS. Statistical significance performed using one-way analysis of variance for multiple comparisons. Mean values not sharing common letters are significantly different (*P*<0.05). Values are shown as mean ± SEM, n=6.

The impairment of intestinal barrier function was evaluated by detecting the levels of diamine oxidase (DAO) and intestinal trefoil factor (ITF) in piglet serum. There was no significant difference in DAO levels among the experimental groups, while the ITF levels were significantly increased in the WT and ΔluxS group than in the Con group (*P*<0.05) (**Figure 3A, B**). In the duodenum, compared with the Con group, the WT group had significantly higher villus height and VCR ratio (*P*<0.05), and had a decrease trend in the crypt depth (*P*=0.07). The VCR ratio significantly increased in the WT group than in the ΔluxS group (*P*<0.05) (**Figure 3C**). In the jejunum, the WT group significantly decreased the crypt depth and improved the VCR ratio compared with the ΔluxS group (*P*<0.05, **Figure 3D**). For the ileum, the VCR ratio in the WT group were significantly higher than that in the Con and ΔluxS groups (*P*<0.05), the villus height in the WT group was significant higher than the ΔluxS group and had an increase trend than the Con group (**Figure 3E**).

We further visualized the inside of the ileum tissue using scanning microscopy (**Figure 3F**). Part of the ileum villi in the Con group was damaged after weaning, while the villus structure was improved in the WT and ΔluxS groups. The denser and more compact ileum microvilli were found in the WT and ΔluxS groups compared that in the Con group. At the same time, expressions of tight junction proteins were detected in ileum tissue of piglet by immunoblotting. Compared with the Con group, the WT group and ΔluxS groups had significantly increased expression of occludin protein (*P*<0.05) (**Figure 3G**). There was no significant difference in the expression of claudin-3 and ZO-1 proteins between the experimental groups (**Figures 3H, I**).

### Colonic goblet cells and mucus secretion

**Figure 4:**
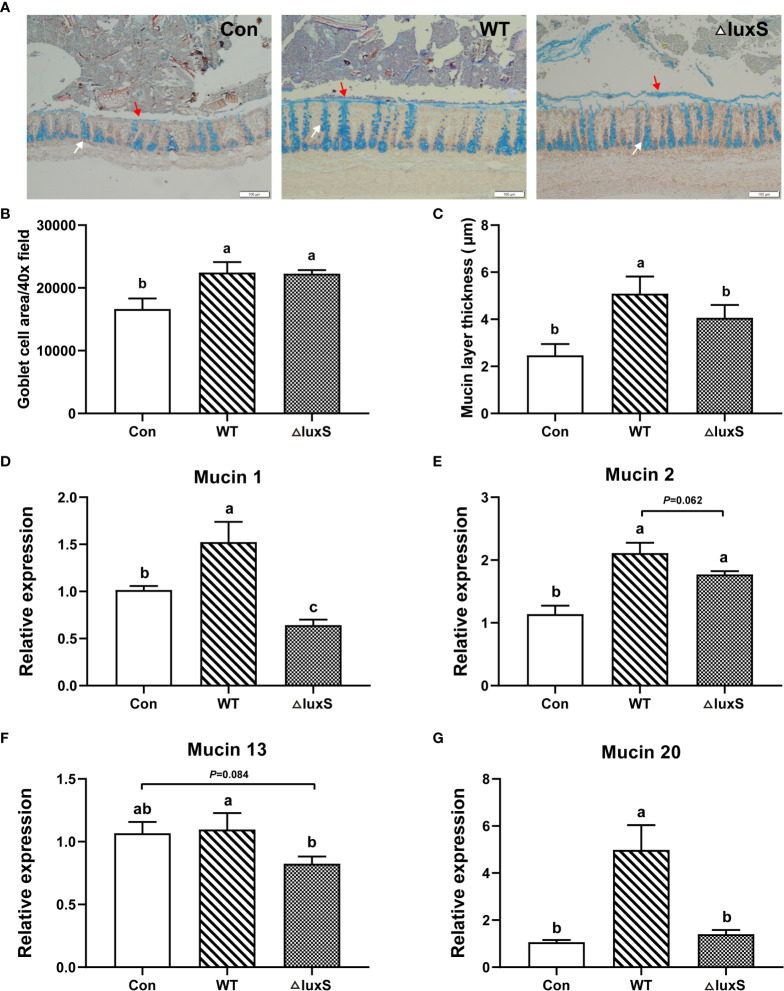
Intestinal chemical barrier functions in weaned piglets. **(A)** Alcian blue staining of colon (100 μm). **(B)** Goblet cell area. **(C)** Mucin layer thickness. Ten fields of view were randomly selected for each sample for statistics. Red arrows point to the mucus layer and white arrows point to goblet cells. **(D-G)** The relative mRNA expression of colonic mucins. GAPDH was used as an internal reference. Each PCR reaction was performed in three technical replicates across six biological replicates. WT and ΔluxS groups were orally inoculated with 2 mL of LGG wild strain and *luxS* mutant strain, respectively, for 3 weeks before weaning on day 21. Con group was orally inoculated with PBS. Statistical significance performed using one-way analysis of variance for multiple comparisons. Mean values not sharing common letters are significantly different (*P*<0.05). Values are shown as mean ± SEM, n=6.

Colonic goblet cells and mucus layer were visualized with alcian blue staining and immunofluorescence, respectively (**Figures 4A–C**). The number of goblet cells in the in the WT and ΔluxS groups was significantly higher than that in the Con group (*P*<0.05). The thickness of the mucus layer in the WT group was significantly higher than that in Con and ΔluxS (*P*<0.05). We further determined the relative mRNA expression of mucin 1, mucin 2, mucin 13 and mucin 20 in colon tissue (**Figures 4D–G**). The relative mRNA expression of intestinal mucins proteins including mucin 1, mucin 2 and mucin 20 were significantly increased in the WT group than in the Con group (*P*<0.05). Meanwhile, the relative mRNA expression of mucin 1, mucin 13 and mucin 20 in the WT group were significantly higher than those in the ΔluxS group (*P*<0.05). The above results demonstrated that feeding of WT could promote the expression of colonic mucin in weaned piglets more than ΔluxS.

### Serum inflammatory factors and antioxidant characteristics

**Figure 5:**
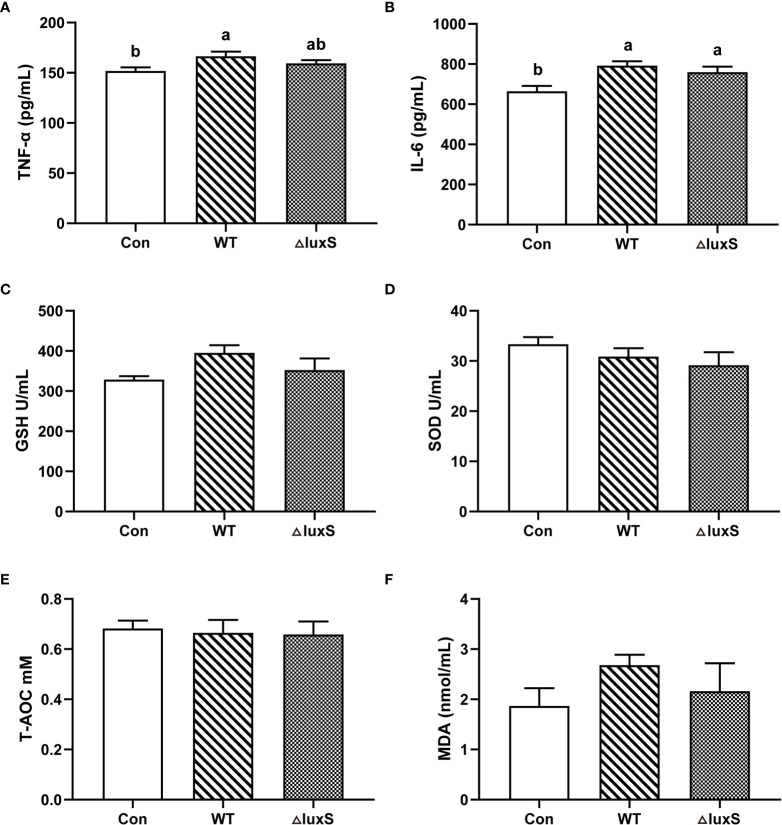
Serum biochemical parameters of weaned piglets. **(A-F)** TNF-α, IL-6, GSH, SOD, T-AOC, MDA, respectively. TNF-α, tumor necrosis factor-α; IL-6, interleukin-6; GSH, glutathione peroxidase; SOD, superoxide dismutase; T-AOC, Total antioxidant capacity; MDA, malonic dialdehyde. WT and ΔluxS groups were orally inoculated with 2 mL of LGG wild strain and *luxS* mutant strain, respectively, for 3 weeks before weaning on day 21. Con group was orally inoculated with PBS. Statistical significance performed using one-way analysis of variance for multiple comparisons. Mean values not sharing common letters are significantly different (*P*<0.05). Values are shown as mean ± SEM, n=6.

The serum inflammatory factors and antioxidant characteristics of piglets after weaning are shown in **Figure 5**. The level of TNF-α in the WT group was significantly higher than the Con group (*P*<0.05), and there was no significant difference in TNF-α level in the ΔluxS group from the Con or WT groups (**Figure 5A**). The expression of IL-6 levels was significantly increased in the WT and ΔluxS groups compared with the Con group (*P*<0.05), while there was not significantly different between the WT and ΔluxS groups (**Figure 5B**). No significant differences were detected in GSH, SOD, T-AOC and MDA concentrations among the experimental groups (**Figures 5C–F**).

### Expression of ileum inflammatory signaling pathways

**Figure 6:**
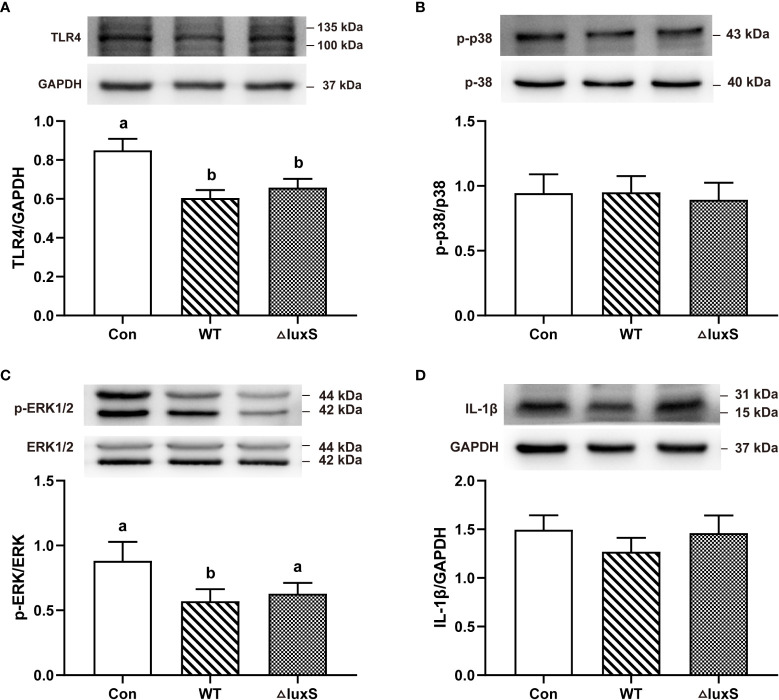
Intestinal immune functions in weaned piglets. **(A-D)** TLR4, p-p38, p-ERK, IL-1β, respectively. The protein expression was quantified by densitometry and normalized to the level of GAPDH, p-38 or ERK1/2, respectively. TLR4, Toll-like receptor 4; p-, phospho-; p38, mitogen-activated protein kinase (MAPK) p38; ERK, extracellular signal-regulated kinase; IL-1β, interleukin-1β. WT and ΔluxS groups were orally inoculated with 2 mL of LGG wild strain and *luxS* mutant strain, respectively, for 3 weeks before weaning on day 21. Con group was orally inoculated with PBS. Statistical significance performed using one-way analysis of variance for multiple comparisons. Mean values not sharing common letters are significantly different (*P*<0.05). Values are shown as mean ± SEM, n=6.

Our experimental results indicated that the addition of WT and ΔluxS did not alter the weight of immune organs indexes, such as spleen and liver (Not shown). We further evaluated the expression of ileum inflammatory signaling pathways by western blot. Compared with the Con group, the expression of TLR4 protein was significantly decreased in the WT and ΔluxS groups (*P*<0.05, **Figures 6A**). The expression of p-ERK protein in the WT group was significantly lower than that in the Con (*P*<0.05, **Figures 6C**). There was no significant difference in the expression levels of p-p38 and IL-1β among the experimental groups (**Figures 6B, D**).

## Discussion

QS system of bacterium has aroused widespread discussion, but less research has been done on the regulation of LuxS/AI-2 QS system on *Lactobacillus* in gut health. In previous studies, we have demonstrated that the production of AI-2 signaling molecules decreased significantly after *luxS* deletion from both qualitative and quantitative perspectives by using BB170 strain and high performance liquid chromatography (HPLC), respectively (15). In this study, we selected newborn piglets with similar body weight and fed LGG or its mutant to them by orally administration immediately after birth. The body weight of piglets fed WT was significantly higher than Con and ΔluxS groups in our study, indicating that WT could promote growth of piglet. Research has showed that piglets fed *Lactobacillus casei* or *Enterococcus faecalis* alone or in combination for 28 days increased daily gain by 18%-27%, which may be related to reduced diarrhea rates and increased nutrient absorption (28).The gastrointestinal tract of animals is colonized with a rich and complex microbial community, and the benign interaction between the immune system and the intestinal microbiota is crucial for maintaining intestinal homeostasis for a lifetime. The gut of piglets is germ-free at birth, and with the intervention of maternal and external microbes, the gut microbiota begins to build. Studies have found that the dominant bacteria in the gastrointestinal tract of piglets are mainly bacteria that can tolerate low pH environment, such as *Lactobacillus spp*, *Streptococcus spp* and *Helicobacter spp* (29). With the increasing age of piglets, the gut will eventually form a homeostasis micro-ecosystem, which helps the animal to improve immunity against various adverse conditions in the outside world. There was no significant difference in α and β diversity between different groups in this study, indicating that supplementation of WT and ΔluxS in advance did not significantly affect the structure of gut microbiota, which may be the result of self-regulation of piglets. Further comparative analysis at the phylum and genus levels, we found that the dominant bacteria in the ileum were mainly *Firmicutes* and *Actinobacteriota*. At the genus level, there was no difference in the relative abundance of *Lactobacillus* in ileal contents among all groups. Combined with the plate counting results, compared with the Con group, the number of *Lactobacillus* in fecal on day 20 was no significant difference between WT and ΔluxS groups. Perhaps this is due to that LGG had been steadily colonized in the gut. We found that supplementation of WT and ΔluxS in advance significantly increased the abundance of *Bifidobacterium* in the piglet gut. It is well known that *Lactobacillus* and *Bifidobacterium*, as important probiotics colonizing the gastrointestinal tract, have functions such as promoting the absorption of nutrients and enhancing immunity, and also has positive significance in the prevention and treatment of diseases (30). *Catenibacterium*, *Eubacterium* and *Lachnospiraceae*, as gut symbiotic bacteria, can regulate the microecological balance of weaned piglets by producing short-chain fatty acid (31). Supplementation of ΔluxS in advance significantly increased the relative abundances of *Catenibacterium*, *Eubacterium*, *Lachnospiraceae* and *Bifidobacterium*, indicating that ΔluxS could increase the proportion of potential probiotics to. QS signaling molecules are lipid-soluble small molecules that easily penetrate cells and affect cell function (32). Bacterial production of the QS signaling molecule N-acyl-homoserine lactone (AHL) in the gut has been shown to affect homeostasis of intestinal epithelial cells (33, 34). At the same time, the level of the signal molecule AI-2 was beneficial to the expansion of the Firmicutes in the antibiotic-treated microbiota, while hindering the Bacteroidetes, indicating that AI-2 counteracted the dysbiosis caused by long-term antibiotic treatment (35). The bacteria in the gut microbiota produce various types of signaling molecules, and the mechanism by which they regulate the behavior of bacteria in the gut under high density environment remains to be further explored.

The integrity of the intestinal morphological structure is the basic condition for all functions to function, and mucosal damage will increase intestinal permeability and the probability of intestinal diseases. Intestinal trefoil factor (ITF) is a small molecule protein secreted by goblet cells on the surface of the intestinal mucosa, which can not only promote the proliferation of intestinal mucosal cells, but also bind to glycoproteins in the mucus. It is important in promoting intestinal repair and maintaining the intestinal mucus barrier (36). It was found that mice overexpressing ITF in the gut had increased resistance to intestinal injury and ulceration, whereas ITF-deficient mice were more susceptible to dextran sulfate sodium (DSS)-induced colitis (37). Our results showed that both WT and ΔluxS supplementation in advance could significantly increase the level of ITF, indicating that LGG maintained the intestinal mucosal barrier and alleviated the gut damage of piglets caused by weaning stress. The normal intestinal morphological structure is the basis of nutrient digestion and absorption, and the development and integrity of intestinal villi directly reflect the degree of intestinal damage. The study confirmed that daily gain and starter conversion had a remarkable increased after feeding piglets with *Lactobacillus johnsonii* BS15, which was mostly ascribe to improved gut development and digestion by ileal villus height and crypt depth (38). Studies also have confirmed that the integrity of the jejunum and ileum villi in piglets at age of 25 days after weaning was better than the control group at 30 and 45 days of age, indicating that LGG effectively protect the normal development of intestinal villi (39). In this study, we found that compared with ΔluxS, WT could better alleviate intestinal damage caused by weaning. We further visualized the ileal villi structure using scanning electron microscopy. We found that weaning stress caused damage to intestinal villi, which changed from finger-like to smooth tongue-like, and sparse microvilli. Supplementation of WT and ΔluxS in advance made the microvilli dense and compact, and significantly alleviated the villus damage. The expression of tight junctions reflects the degree of connection between epithelial cells and the integrity of the intestinal barrier function. *Lactobacillus reuteri* LR1 increased the expression of the tight junction proteins ZO-1 and occludin in the jejunum and ileum of weaned piglets, as well as the expression of Muc2 in the ileal mucosa (40). Our results show that WT and ΔluxS could maintain intestinal barrier function mainly by promoting the expression of occludin protein. The mucus layer plays critical roles in protecting intestinal barrier, and the loss of the mucin layer can also affect the evolution of dysbiosis (41). In experimental models of *Pseudomonas Aeruginosa Pneumonia*, LGG can maintain intestinal barrier homeostasis by promoting mucin secretion and layer formation, as well as increasing the expression of Muc2 (42). In this study, WT and ΔluxS promoted the growth and development of colonic goblet cells. However, the ability of ΔluxS to produce and secrete mucus significantly decreased compared to WT. A recent study found that Atlantic salmon mucins reduce AI-2 secretion of *A. salmonicidal* in a N-acetylneuraminic acid (NeuAc-) and luxS-dependent manner (43). Combined with our previous experiments, the ability of interbacterial adhesion and biofilm formation significantly decreased in *luxS* mutant. At the same time, the ability of luxS-deficient strains to colonize the germ-free zebrafish significantly decreased. So we speculated that the LuxS/AI-2 QS system might be involved in the adhesion of *Lactobacillus* to intestine and to regulate the secretion of intestinal mucus.

Impaired gut barrier function often provokes an inflammatory response, leading to activation of systemic immunity and production of pro-inflammatory cytokines (44). LGG has a two-way immune regulation to maintain the homeostasis of gut immune function by regulating the expression of cytokines. Treatment of healthy IPEC-J2 cells with LGG promoted the production of IL-6, whereas LGG treated rotavirus infection by reducing the level of IL-6 (45). It can be seen that LGG has different immunomodulatory effects under different stimulation conditions. Inflammatory factors can bind to the membrane receptor TLR4, then trigger the TLR4/NF-κB signaling pathway to secrete a large number of inflammatory cytokines, and eventually lead to tissue damage (46). LGG ameliorated DON- and LPS-induced inflammation by modulating the TLR4/NF-κB signaling pathway (47, 48). A recent report showed a new soluble protein from LGG, HM0539, suppress inflammation by inhibiting the TLR4/MyD88/NF-κB signaling, which is considered as a potential therapeutic option for inflammatory bowel disease (IBD) (49). Our results also showed that supplementation of WT and ΔluxS in advance down-regulated the expression of TLR4 and p-ERK pathways, but significantly increased the expression of TNF-α and IL-6 pro-inflammatory factors in serum. On the one hand, the whole release of inflammatory cytokines in the serum may be responsible for chronic inflammation (50). On the other hand, LGG was fed to the piglets at the interval of birth in our experiment. LGG is an exogenous antigen for newborn piglets, which can induce the signal transduction of inflammatory factors in the intestine and stimulate the body to produce a certain amount of pro-inflammatory factors. The probiotic properties of LGG are tolerated in the gut, so that the secretion of pro-inflammatory factors is maintained at a level that can both enhance the gut’s defense against foreign microbial invasion and reduce self-stimulation to the piglets.

## Conclusions

Both the wild and the luxS-deficient strain could restore the gut microbiota, gut barrier function and immune regulation in weaned piglet. But the ability of ΔluxS to maintain intestinal morphology and promote mucus secretion from goblet cells was significantly decreased.

## Data availability statement

The datasets presented in this study can be found in online repositories. The names of the repository/repositories and accession number(s) can be found below: https://www.ncbi.nlm.nih.gov/, PRJNA856449.

## Ethics statement

The animal study was reviewed and approved by Animal Care and Use Committee of Zhejiang University (ETHICS CODE Permit no. ZJU20170529).

## Author contributions

HW and ZD conceived the study. ZD, JD, YW, YFM, YYM, JZ and WH performed the experiments, analyzed the data or provided the support for the trial. HW and ZD interpreted the data and wrote the manuscript. All authors contributed to the article and approved the submitted version.

## References

[1] Wu Y Zhao J Xu C Ma N He T Zhao J . Progress towards pig nutrition in the last 27 years. J Sci Food Agric (2020) 100:5102–10. doi: 10.1002/jsfa.9095 29691867

[2] Deng Z Hou K Zhao J Wang H . The probiotic properties of lactic acid bacteria and their applications in animal husbandry. Curr Microbiol (2021) 79:22. doi: 10.1007/s00284-021-02722-3 34905106

[3] Smith F Clark JE Overman BL Tozel CC Huang JH Rivier JE . Early weaning stress impairs development of mucosal barrier function in the porcine intestine. Am J Physiol Gastrointest liver Physiol (2010) 298:G352–63. doi: 10.1152/ajpgi.00081.2009 PMC2838512 19926814

[4] Jayaraman B Nyachoti CM . Husbandry practices and gut health outcomes in weaned piglets: A review. Anim Nutr (Zhongguo xu mu shou yi xue hui) (2017) 3:205–11. doi: 10.1016/j.aninu.2017.06.002 PMC5941228 29767154

[5] Yan F Polk DB . Probiotic bacterium prevents cytokine-induced apoptosis in intestinal epithelial cells. J Biol Chem (2002) 277:50959–65. doi: 10.1074/jbc.M207050200 PMC4006994 12393915

[6] Chen RC Xu LM Du SJ Huang SS Wu H Dong JJ . Lactobacillus rhamnosus GG supernatant promotes intestinal barrier function, balances treg and TH17 cells and ameliorates hepatic injury in a mouse model of chronic-binge alcohol feeding. Toxicol Lett (2016) 241:103–10. doi: 10.1016/j.toxlet.2015.11.019 26617183

[7] Mao X Gu C Hu H Tang J Chen D Yu B . Dietary lactobacillus rhamnosus GG supplementation improves the mucosal barrier function in the intestine of weaned piglets challenged by porcine rotavirus. PloS One (2016) 11:e0146312. doi: 10.1371/journal.pone.0146312 26727003 PMC4699646

[8] Okumura R Takeda K . Roles of intestinal epithelial cells in the maintenance of gut homeostasis. Exp Mol Med (2017) 49:e338. doi: 10.1038/emm.2017.20 28546564 PMC5454438

[9] Kelly JR Kennedy PJ Cryan JF Dinan TG Clarke G Hyland NP . Breaking down the barriers: The gut microbiome, intestinal permeability and stress-related psychiatric disorders. Front Cell Neurosci (2015) 9:392. doi: 10.3389/fncel.2015.00392 26528128 PMC4604320

[10] Turner JR . Intestinal mucosal barrier function in health and disease. Nat Rev Immunol (2009) 9:799–809. doi: 10.1038/nri2653 19855405

[11] Lee SH . Intestinal permeability regulation by tight junction: Implication on inflammatory bowel diseases. Intestinal Res (2015) 13:11–8. doi: 10.5217/ir.2015.13.1.11 PMC4316216 25691839

[12] Johansson ME Hansson GC . Immunological aspects of intestinal mucus and mucins. Nat Rev Immunol (2016) 16:639–49. doi: 10.1038/nri.2016.88 PMC6435297 27498766

[13] Han X Lee A Huang S Gao J Spence JR Owyang C . *Lactobacillus rhamnosus* GG prevents epithelial barrier dysfunction induced by interferon-gamma and fecal supernatants from irritable bowel syndrome patients in human intestinal enteroids and colonoids. Gut Microbes (2019) 10:59–76. doi: 10.1080/19490976.2018.1479625 30040527 PMC6363076

[14] Yi L Dong X Grenier D Wang K Wang Y . Research progress of bacterial quorum sensing receptors: Classification, structure, function and characteristics. Sci total Environ (2021) 763:143031. doi: 10.1016/j.scitotenv.2020.143031 33129525

[15] Deng Z Hou K Valencak TG Luo XM Liu J Wang H . AI-2/LuxS quorum sensing system promotes biofilm formation of lactobacillus rhamnosus GG and enhances the resistance to enterotoxigenic escherichia coli in germ-free zebrafish. Microbiol Spectr (2022) 10(4):e0061022. doi: 10.1128/spectrum.00610-22 35700135 PMC9430243

[16] Bivar Xavier K . Bacterial interspecies quorum sensing in the mammalian gut microbiota. Comptes Rendus Biol (2018) 341:297–9. doi: 10.1016/j.crvi.2018.03.006 29631889

[17] Takahashi S Tomita J Nishioka K Hisada T Nishijima M . Development of a prokaryotic universal primer for simultaneous analysis of bacteria and archaea using next-generation sequencing. PloS One (2014) 9:e105592. doi: 10.1371/journal.pone.0105592 25144201 PMC4140814

[18] Parikh HI Koparde VN Bradley SP Buck GA Sheth NU . MeFiT: Merging and filtering tool for illumina paired-end reads for 16S rRNA amplicon sequencing. BMC Bioinf (2016) 17:491. doi: 10.1186/s12859-016-1358-1 PMC5134250 27905885

[19] Chen S Zhou Y Chen Y Gu J . Fastp: An ultra-fast all-in-one FASTQ preprocessor. Bioinformatics (2018) 34:i884–90. doi: 10.1093/bioinformatics/bty560 PMC6129281 30423086

[20] Magoč T Salzberg SL . FLASH: Fast length adjustment of short reads to improve genome assemblies. Bioinformatics (2011) 27:2957–63. doi: 10.1093/bioinformatics/btr507 PMC3198573 21903629

[21] Edgar RC . UPARSE: highly accurate OTU sequences from microbial amplicon reads. Nat Methods (2013) 10:996–8. doi: 10.1038/nmeth.2604 23955772

[22] Nabuurs MJ Hoogendoorn A van der Molen EJ van Osta AL . Villus height and crypt depth in weaned and unweaned pigs, reared under various circumstances in the Netherlands. Res vet Sci (1993) 55:78–84. doi: 10.1016/0034-5288(93)90038-H 8378616

[23] Ludikhuize MC Meerlo M Gallego MP Xanthakis D Burgaya Julià M Nguyen NTB . Mitochondria define intestinal stem cell differentiation downstream of a FOXO/Notch axis. Cell Metab (2020) 32:889–900.e7. doi: 10.1016/j.cmet.2020.10.005 33147486

[24] Riva A Kuzyk O Forsberg E Siuzdak G Pfann C Herbold C . A fiber-deprived diet disturbs the fine-scale spatial architecture of the murine colon microbiome. Nat Commun (2019) 10:4366. doi: 10.1038/s41467-019-12413-0 31554820 PMC6761162

[25] Desai MS Seekatz AM Koropatkin NM Kamada N Hickey CA Wolter M . A dietary fiber-deprived gut microbiota degrades the colonic mucus barrier and enhances pathogen susceptibility. Cell (2016) 167:1339–1353.e21. doi: 10.1016/j.cell.2016.10.043 27863247 PMC5131798

[26] Wang Y Wu Y Wang Y Fu A Gong L Li W . Bacillus amyloliquefaciens SC06 alleviates the oxidative stress of IPEC-1 *via* modulating Nrf2/Keap1 signaling pathway and decreasing ROS production. Appl Microbiol Biotechnol (2017) 101:3015–26. doi: 10.1007/s00253-016-8032-4 27957629

[27] Cui Y Liu L Dou X Wang C Zhang W Gao K . Lactobacillus reuteri ZJ617 maintains intestinal integrity *via* regulating tight junction, autophagy and apoptosis in mice challenged with lipopolysaccharide. Oncotarget (2017) 8:77489–99. doi: 10.18632/oncotarget.20536 PMC5652795 29100403

[28] Liu C Zhu Q Chang J Yin Q Song A Li Z . Effects of *lactobacillus casei* and *enterococcus faecalis* on growth performance, immune function and gut microbiota of suckling piglets. Arch Anim Nutr (2017) 71:120–33. doi: 10.1080/1745039X.2017.1283824 28201936

[29] Jensen AR Elnif J Burrin DG Sangild PT . Development of intestinal immunoglobulin absorption and enzyme activities in neonatal pigs is diet dependent. J Nutr (2001) 131:3259–65. doi: 10.1093/jn/131.12.3259 11739877

[30] Vlasova AN Kandasamy S Chattha KS Rajashekara G Saif LJ . Comparison of probiotic lactobacilli and bifidobacteria effects, immune responses and rotavirus vaccines and infection in different host species. Vet Immunol immunopathol (2016) 172:72–84. doi: 10.1016/j.vetimm.2016.01.003 26809484 PMC4818210

[31] Dadi TH Vahjen W Zentek J Melzig MF Granica S Piwowarski JP . Lythrum salicaria l. herb and gut microbiota of healthy post-weaning piglets. focus on prebiotic properties and formation of postbiotic metabolites in *ex vivo* cultures. J ethnopharmacol (2020) 261:113073. doi: 10.1016/j.jep.2020.113073 32673710

[32] Kendall MM Sperandio V . What a dinner party! mechanisms and functions of interkingdom signaling in host-pathogen associations. mBio (2016) 7:e01748. doi: 10.1128/mBio.01748-15 26933054 PMC4810492

[33] Tao S Sun Q Cai L Geng Y Hua C Ni Y . Caspase-1-dependent mechanism mediating the harmful impacts of the quorum-sensing molecule n-(3-oxo-dodecanoyl)-l-homoserine lactone on the intestinal cells. J Cell Physiol (2019) 234:3621–33. doi: 10.1002/jcp.27132 30471106

[34] Landman C Grill JP Mallet JM Marteau P Humbert L Le Balc'h E . Inter-kingdom effect on epithelial cells of the n-acyl homoserine lactone 3-oxo-C12:2, a major quorum-sensing molecule from gut microbiota. PloS One (2018) 13:e0202587. doi: 10.1371/journal.pone.0202587 30157234 PMC6114859

[35] Thompson JA Oliveira RA Djukovic A Ubeda C Xavier KB . Manipulation of the quorum sensing signal AI-2 affects the antibiotic-treated gut microbiota. Cell Rep (2015) 10:1861–71. doi: 10.1016/j.celrep.2015.02.049 25801025

[36] Uchino H Kataoka H Itoh H Hamasuna R Koono M . Overexpression of intestinal trefoil factor in human colon carcinoma cells reduces cellular growth *in vitro* and *in vivo* . Gastroenterology (2000) 118(1):60–9. doi: 10.1016/s0016-5085(00)70414-8 10611154

[37] Kim YS Ho SB . Intestinal goblet cells and mucins in health and disease: recent insights and progress. Curr Gastroenterol Rep (2010) 12:319–30. doi: 10.1007/s11894-010-0131-2 PMC2933006 20703838

[38] Xin J Zeng D Wang H Sun N Zhao Y Dan Y . Probiotic *lactobacillus johnsonii* BS15 promotes growth performance, intestinal immunity, and gut microbiota in piglets. Probiotics antimicrobial Proteins (2020) 12:184–93. doi: 10.1007/s12602-018-9511-y 30617949

[39] Shonyela SM Feng B Yang W Yang G Wang C . The regulatory effect of lactobacillus rhamnosus GG on T lymphocyte and the development of intestinal villi in piglets of different periods. AMB Express (2020) 10:76. doi: 10.1186/s13568-020-00980-1 32303860 PMC7165236

[40] Yi H Wang L Xiong Y Wen X Wang Z Yang X . Effects of *lactobacillus reuteri* LR1 on the growth performance, intestinal morphology, and intestinal barrier function in weaned pigs. J Anim Sci (2018) 96:2342–51. doi: 10.1093/jas/sky129 PMC6095392 29659876

[41] Chen SJ Liu XW Liu JP Yang XY Lu FG . Ulcerative colitis as a polymicrobial infection characterized by sustained broken mucus barrier. World J Gastroenterol (2014) 20:9468–75. doi: 10.3748/wjg.v20.i28.9468 PMC4110578 25071341

[42] Khailova L Baird CH Rush AA Barnes C Wischmeyer PE . Lactobacillus rhamnosus GG treatment improves intestinal permeability and modulates inflammatory response and homeostasis of spleen and colon in experimental model of pseudomonas aeruginosa pneumonia. Clin Nutr (Edinburgh Scotland) (2017) 36:1549–57. doi: 10.1016/j.clnu.2016.09.025 PMC5641477 27745813

[43] Padra JT Loibman SO Thorell K Sundh H Sundell K Lindén SK . Atlantic Salmon mucins inhibit LuxS-dependent a. salmonicida AI-2 quorum sensing in an n-acetylneuraminic acid-dependent manner. Int J Mol Sci (2022) 23:4326. doi: 10.3390/ijms23084326 35457143 PMC9026418

[44] Chelakkot C Ghim J Ryu SH . Mechanisms regulating intestinal barrier integrity and its pathological implications. Exp Mol Med (2018) 50:1–9. doi: 10.1038/s12276-018-0126-x PMC6095905 30115904

[45] Liu F Li G Wen K Bui T Cao D Zhang Y . Porcine small intestinal epithelial cell line (IPEC-J2) of rotavirus infection as a new model for the study of innate immune responses to rotaviruses and probiotics. Viral Immunol (2010) 23:135–49. doi: 10.1089/vim.2009.0088 PMC2883522 20373994

[46] Han JM Lee EK Gong SY Sohng JK Kang YJ Jung HJ . Sparassis crispa exerts anti-inflammatory activity *via* suppression of TLR-mediated NF-κB and MAPK signaling pathways in LPS-induced RAW264.7 macrophage cells. J ethnopharmacol (2019) 231:10–8. doi: 10.1016/j.jep.2018.11.003 30395976

[47] Mao J Qi S Cui Y Dou X Luo XM Liu J . *Lactobacillus rhamnosus* GG attenuates lipopolysaccharide-induced inflammation and barrier dysfunction by regulating MAPK/NF-κB signaling and modulating metabolome in the piglet intestine. J Nutr (2020) 150:1313–23. doi: 10.1093/jn/nxaa009 32027752

[48] Bai Y Ma K Li J Ren Z Zhang J Shan A . Lactobacillus rhamnosus GG ameliorates DON-induced intestinal damage depending on the enrichment of beneficial bacteria in weaned piglets. J Anim Sci Biotechnol (2022) 13:90. doi: 10.1186/s40104-022-00737-9 35962456 PMC9375241

[49] Li Y Yang S Lun J Gao J Gao X Gong Z . Inhibitory effects of the lactobacillus rhamnosus GG effector protein HM0539 on inflammatory response through the TLR4/MyD88/NF-кB axis. Front Immunol (2020) 11:551449. doi: 10.3389/fimmu.2020.551449 PMC7573360 33123130

[50] Zhou X Zhang Y He L Wan D Liu G Wu X . Serine prevents LPS-induced intestinal inflammation and barrier damage *via* p53-dependent glutathione synthesis and AMPK activation. J Funct Foods (2017) 39:225–32. doi: 10.1016/j.jff.2017.10.026

