# Supplementary material for: Comparison between Lactobacillus rhamnosus GG and LuxS-deficient strain in regulating gut barrier function and inflammation in early-weaned piglets
Source: Front Immunol. 2022 Dec 8;13:1080789. doi: 10.3389/fimmu.2022.1080789 (PMC9773554; doi:10.3389/fimmu.2022.1080789)
Supplement: Supplementary file 1 [file Table_1.docx]

**Supplementary Table S1**

| **Supplementary Table S1. Primer sequences for RT-qPCR** | | |
| --- | --- | --- |
| **Primers** | **5'-3'** | **Sequence** |
| **GAPDH** | Forward | CCTTCATTGACCTCCACTACAT |
|  | Reverse | GGATCTCGCTCCTGGAAGA |
| **Mucin 1** | Forward | GTGCCGACGAAAGAACTG |
|  | Reverse | TGCCAGGTTCGAGTAAGAG |
| **Mucin 2** | Forward | CTGTGTGGGGCCTGACAA |
|  | Reverse | AGTGCTTGCAGTCGAACTCA |
| **Mucin 13** | Forward | GCTACAGTGGAGTTGGCTGT |
|  | Reverse | GACGAATGCAATCACCAGGC |
| **Mucin 20** | Forward | AGGCAGTTACAACATCCACAGAAG |
|  | Reverse | CTGTAGACCATGGCCGAGAAC |
